# Drug voyager: a computational platform for exploring unintended drug action

**DOI:** 10.1186/s12859-017-1558-3

**Published:** 2017-02-28

**Authors:** Min Oh, Jaegyoon Ahn, Taekeon Lee, Giup Jang, Chihyun Park, Youngmi Yoon

**Affiliations:** 1 Department of Computer Science, Virginia Tech, Blacksburg, VA USA; 2 Department of Computer Science & Engineering, Incheon National University, Incheon, South Korea; 3 Department of Computer Engineering, Gachon University, Seongnam, South Korea; 4 Biomedical HPC Technology Research Center, Korean Institute of Science and Technology Information, Daejeon, South Korea; 5 Postal Address: Gachon University, 339Ho, Woongji B.D., 1324 Seongnam-daero, Seongnam-si, 13120 South Korea

**Keywords:** Drug pathway, Drug-signaling pathway, Drug action, Pharmacodynamics, Drug repurposing, Drug repositioning, Adverse reactions, Side effects

## Abstract

**Background:**

The dominant paradigm in understanding drug action focuses on the intended therapeutic effects and frequent adverse reactions. However, this approach may limit opportunities to grasp unintended drug actions, which can open up channels to repurpose existing drugs and identify rare adverse drug reactions. Advances in systems biology can be exploited to comprehensively understand pharmacodynamic actions, although proper frameworks to represent drug actions are still lacking.

**Results:**

We suggest a novel platform to construct a drug-specific pathway in which a molecular-level mechanism of action is formulated based on pharmacologic, pharmacogenomic, transcriptomic, and phenotypic data related to drug response (http://databio.gachon.ac.kr/tools/). In this platform, an adoption of three conceptual levels imitating drug perturbation allows these pathways to be realistically rendered in comparison to those of other models. Furthermore, we propose a new method that exploits functional features of the drug-specific pathways to predict new indications as well as adverse reactions. For therapeutic uses, our predictions significantly overlapped with clinical trials and an up-to-date drug-disease association database. Also, our method outperforms existing methods with regard to classification of active compounds for cancers. For adverse reactions, our predictions were significantly enriched in an independent database derived from the Food and Drug Administration (FDA) Adverse Event Reporting System and meaningfully cover an Adverse Reaction Database provided by Health Canada. Lastly, we discuss several predictions for both therapeutic indications and side-effects through the published literature.

**Conclusions:**

Our study addresses how we can computationally represent drug-signaling pathways to understand unintended drug actions and to facilitate drug discovery and screening.

**Electronic supplementary material:**

The online version of this article (doi:10.1186/s12859-017-1558-3) contains supplementary material, which is available to authorized users.

## Background

The actions of drugs have been systematically observed and recorded by governments, non-trading organizations, and academic institutions. From phenotypic screening to post-marketing surveillance, abundant reports have been archived and follow-up studies on the mechanisms of action of drugs have been conducted. Although this research delivers us advances in knowledge, our understanding of drug actions is generally biased toward intended therapeutic effects and frequent adverse reactions. This partiality has caused delays in deciphering the mechanisms of unintended drug actions. Historically, it was inevitable that the discovery of unexpected drug actions, regardless of whether they are desirable or not, usually depends on empirical detection [1–3]. However, an unbiased analysis of drug actions should be a basis for understanding unintended drug responses and predicting drug-repositioning opportunities or undesirable reactions.

The rapidly expanding databases and newly available data in the literature, including pharmacogenomic biomarkers, drug-induced gene expression profiles, and drug side-effect information, continually provide clues which indicate unknown drug actions [1, 3, 4]. Recently, computational approaches for systematic analyses of these data have been highlighted, enhancing both the availability and usability of the data [4]. In comparison to in vitro and in vivo experiments, computational approaches are remarkable in terms of time and cost efficiency. Moreover, systematic implementations are reproducible. These implementations can be utilized for upcoming drugs as well as failed drugs, but a lack of appropriate methods creates an arduous task for those who attempt to integrate and utilize these scattered pieces of evidence.

For a comprehensive understanding of drug action, it is necessary to organize and analyze drug-signaling pathways in a systematic manner. There have been many attempts to predict drug actions based on similar properties of drugs, including their targets, chemical structures and side effects [5, 6]. Although these properties are fairly useful for distinguishing repurposed drugs, these attempts tend to depend on the extrinsic properties of drugs and not on the intrinsic mechanisms of drug actions. Therefore, the findings are limited. One of the most tangible mechanisms of action is a network in which the nodes refer to biomolecules and the edges refer to the physical interaction between two nodes [7]. It should be noted that drugs exert their effects through multiple signaling cascades in a molecular network rather than through a single gene or a single route. Therefore, we need to devise a network platform which realistically infers the drug-signaling pathways.

Previously, few methods attempted to design drug-signaling pathways at the molecular level in order to identify a novel pathway for a particular drug [8, 9]. However, these methods tended to utilize limited resources to generate the pathway or do not consider the directionality of biological networks. Moreover, systematic approaches to represent the perturbation of molecular and cellular responses are lacking, as the field is in its infancy.

**Fig. 1:**
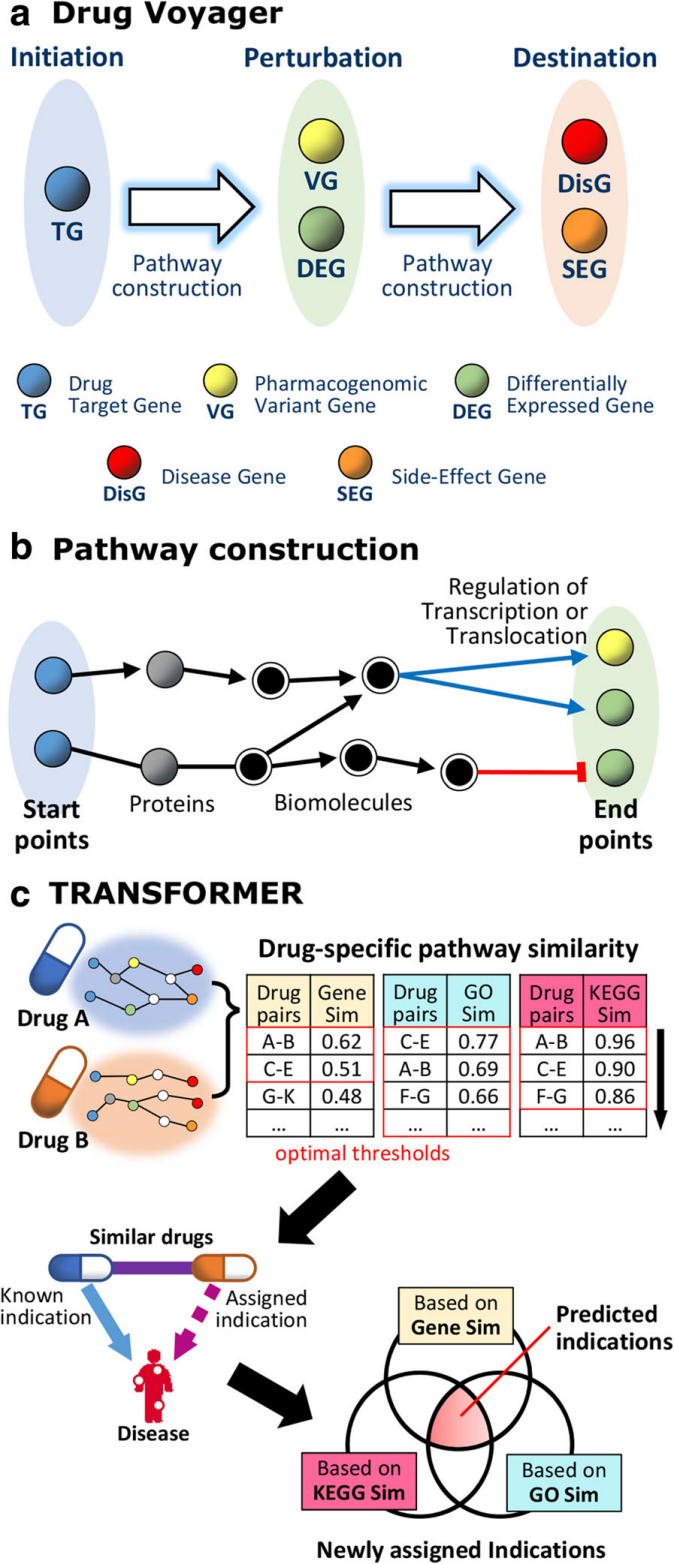
Method overview: **a** Drug Voyager representing the molecular-level actions of drugs by connecting three conceptual levels; **b** constructing a pathway from the starting point to the end point; and **c** TRANSFORMER, utilizing drug-specific pathways to predict drug indications and adverse reactions

Here, we devise a novel platform, called Drug Voyager (http://databio.gachon.ac.kr/tools/), on which to construct drug-signaling pathways for different drugs (Fig. 1). With this platform, the molecular-level action of a drug is represented by connecting the three conceptual levels of “initiation,” “perturbation,” and “destination.” Each level includes a combination of the five types of seed genes related to drug responses and phenotypes: drug target genes (TG), pharmacogenomic variant genes (VG), differentially expressed genes (DEG), disease genes (DisG), and side-effect genes (SEG). As a consequence of construction of level-to-level pathways, 82 drug-signaling pathways were generated in total for 82 drugs. In the validation step, these pathways were significantly enriched in known drug pathway databases and show higher significance levels compared to when other models are used.

Furthermore, we present TRANSFORMER, a new method for **trans**lating the **f**unctional features **o**f d**r**ug-signaling pathways into new **me**dicine and adverse **r**eactions. Based on the drug-signaling pathways generated by Drug Voyager and a snapshot of the cross-talk within each pathway, TRANSFORMER predicts drug indications and adverse reactions. Our predictions for therapeutic use significantly overlaps with drug indications currently tested in clinical trials and an up-to-date drug-disease association database. When used to predict PubChem bioassay results, TRANSFORMER surpasses existing methods in terms of its ability to classify active compounds for cancers. For adverse reactions, our predictions show high significance levels in enrichment testing for an independent database derived from Adverse Event Reporting System [10] of the Food and Drug Administration (FDA) and meaningfully cover the Adverse Reaction Database provided from Health Canada [11]. Lastly, we highlight several predictions for both therapeutic indications and side effects through pathway analyses and from published reports.

## Results

### Validation of drug-signaling pathways

As noted above, we devised a novel platform, termed Drug Voyager, which constructs drug-signaling pathways for individual drugs. In this platform, the three conceptual levels of initiation, perturbation, and destination were adopted to imitate drug perturbations. We assume that a signal transduction cascade in drug action begins at the initiation level and reaches the destination level through the perturbation level. Given the genes that contribute to drug responses and phenotypes, they were assigned to corresponding levels and the mechanism of action of a drug was delineated by connecting these three conceptual levels (Methods section). Among all the FDA approved drugs, some drugs whose corresponding genes are unknown have been filtered out, and finally 82 drugs remain. By constructing level-to-level pathways, Drug Voyager built 82 drug-signaling pathways, one for each of 82 drugs. The validity of each of these pathways was evaluated by the following three observations: 1) enrichment in the Small Molecule Pathway Database (SMPDB) [12], 2) enrichment in PharmGKB [13], and 3) the co-occurrence of the drug and genes in the literature. We then compared Drug Voyager with other pathway construction models.

We undertook gene enrichment computations in the curated drug-action pathways extracted from SMPDB [12]. The drug pathways from SMPDB were generated based on various medical and pharmacology textbooks, as well as relevant published reviews and online databases such as KEGG [14] and the Medical Biochemistry Page [15]. Among the 82 drugs, information about the drug-action pathways was accessible for 35, and we concentrated on 25 of these which had pathways composed of ten or more elements. Twenty-one (84%) of the 25 drug-signaling pathways were significantly enriched in the curated drug-action pathways (one-tailed Fisher’s exact *P* < 0.05).

In addition, the validity of the drug-signaling pathways was tested by comparing these pathways to the pharmacodynamic pathways derived from PharmGKB [13]. Pharmacodynamic pathways depict the pharmacodynamics of a drug based on evidence obtained through an extensive review of a variety of sources, including the U.S. FDA biomarker list [16] and Clinical Pharmacogenetics Implementation Consortium (CPIC) nominations [17]. Out of the 82 drugs, it was possible to obtain pharmacodynamic pathways from PharmGKB for three: valproic acid, methotrexate, and etoposide. All three drug-signaling pathways derived from Drug Voyager for these drugs were significantly enriched in pharmacodynamic pathways in terms of their member genes (one-tailed Fisher’s exact P = 9.27E-09, 3.79E-10, and 7.55E-07, respectively).

Furthermore, by querying PubMed, we counted the co-occurrences of a drug and member genes for each drug-signaling pathway in the literature. The public application program interface (API) of the National Center for Biotechnology Information (NCBI), E-utilities, was used to send queries, with each query made up of a drug generic name and an official gene symbol. For comparison to a random control (the same drug and random gene queries), the seed genes, which were used to construct the pathway, were removed from the pathway members, and a random control was generated with an identical number of the remaining member genes. We found that pathway members for each drug significantly co-occurred when they were compared to random controls (81/82 drugs, Wilcoxon rank sum *P* < 0.05).

We compared the significance of Drug Voyager with the significance levels of three other models: 1) the basic model, 2) the Silberberg model [8], and 3) the Gottlieb model [9]. Our platform, Drug Voyager, employs the three conceptual levels of initiation, perturbation, and destination, and it assigns drug-specific genes to these levels. In contrast, the basic model is a simple model that utilizes the same genes used by Drug Voyager, but at two levels. This model consists of a start level, which contains TGs, and an end level, which involves the other four types of genes (VG, DEG, DisG, and SEG). With this model, the pathway for each drug was constructed using the same construction method used with Drug Voyager.

**Fig. 2:**
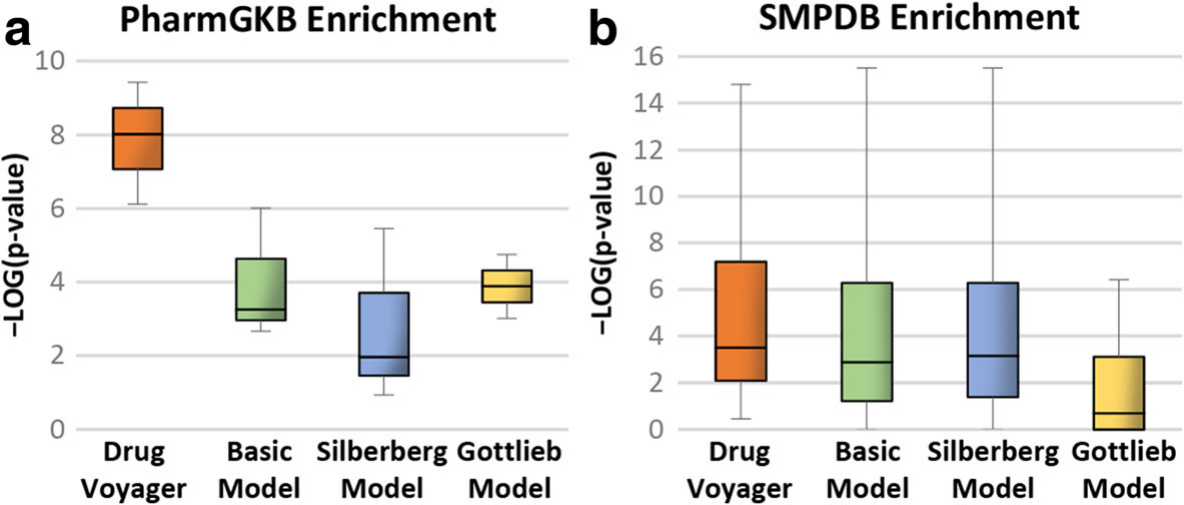
Enrichment comparison with other models: **a** PharmGKB enrichment tests and **b** SMPDB enrichment tests for the drug pathways of each model

Silberberg et al. [8] reconstructed drug-specific subnetworks for each drug by connecting TGs to DEGs. More recently, the Gottlieb model [9] applied three types of genes (TG, VG, and DisG) and linked them to each other to build drug-specific pathways for each drug. For comparison, we used their models with up-to-date interaction networks equivalent to Drug Voyager. Figures 2a, b display box plots of the enrichment tests for each model applied to the same set of drugs and show that the pathway members of our model were more significantly enriched than those of the other methods in the curated drug pathways.

### Drug-signaling pathway as a better indicator of drug repositioning

**Fig. 3:**
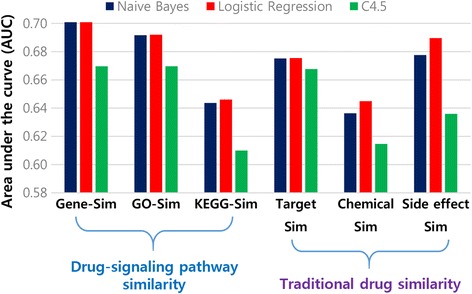
Drug similarity-based classification results: AUC values for drug-similarity-based classifications based on the drug-signaling pathway similarity and traditional drug-similarity measures. Drug-signaling pathway similarity includes the gene component similarity (Gene-Sim), gene ontology enrichment similarity (GO-Sim) and KEGG enrichment similarity (KEGG-Sim). Traditional drug similarity involves drug target similarity (Target Sim), chemical similarity (Chemical Sim) and side-effect similarity (Side effect Sim)

Drug repositioning has been of great interest to the pharmaceutical industry, with increased numbers of systematic analyses to identify additional drug indications [4]. Similar properties of drugs have been used to generate new hypotheses on drug indications and have been considered the crucial basis for computational drug-repositioning approaches [5, 6]. We harness drug-signaling pathways to calculate drug similarity levels and compare the results with prime similarity measurements to discriminate known chemical-disease associations [18] from unknown chemical-disease pairs. The similarity levels of drug-signaling pathways were computed in three different ways: Gene-Sim, GO-Sim and KEGG-Sim (Method section). Other similarity measures traditionally successful in predicting drug indications were compiled, i.e., chemical similarity, drug target similarity and side-effect similarity measures (Additional file 1). Based on each similarity measure, feature values were assigned to each drug indication and classifiers learned using several classification algorithms, including Naive Bayes, Logistic Regression and Decision Tree (C4.5). We found that classifiers using the drug-signaling pathway similarity computed by Gene-Sim and GO-Sim show better performance than those using other similarity measures when predicting known drug associations (Fig. 3).

**Fig. 4:**
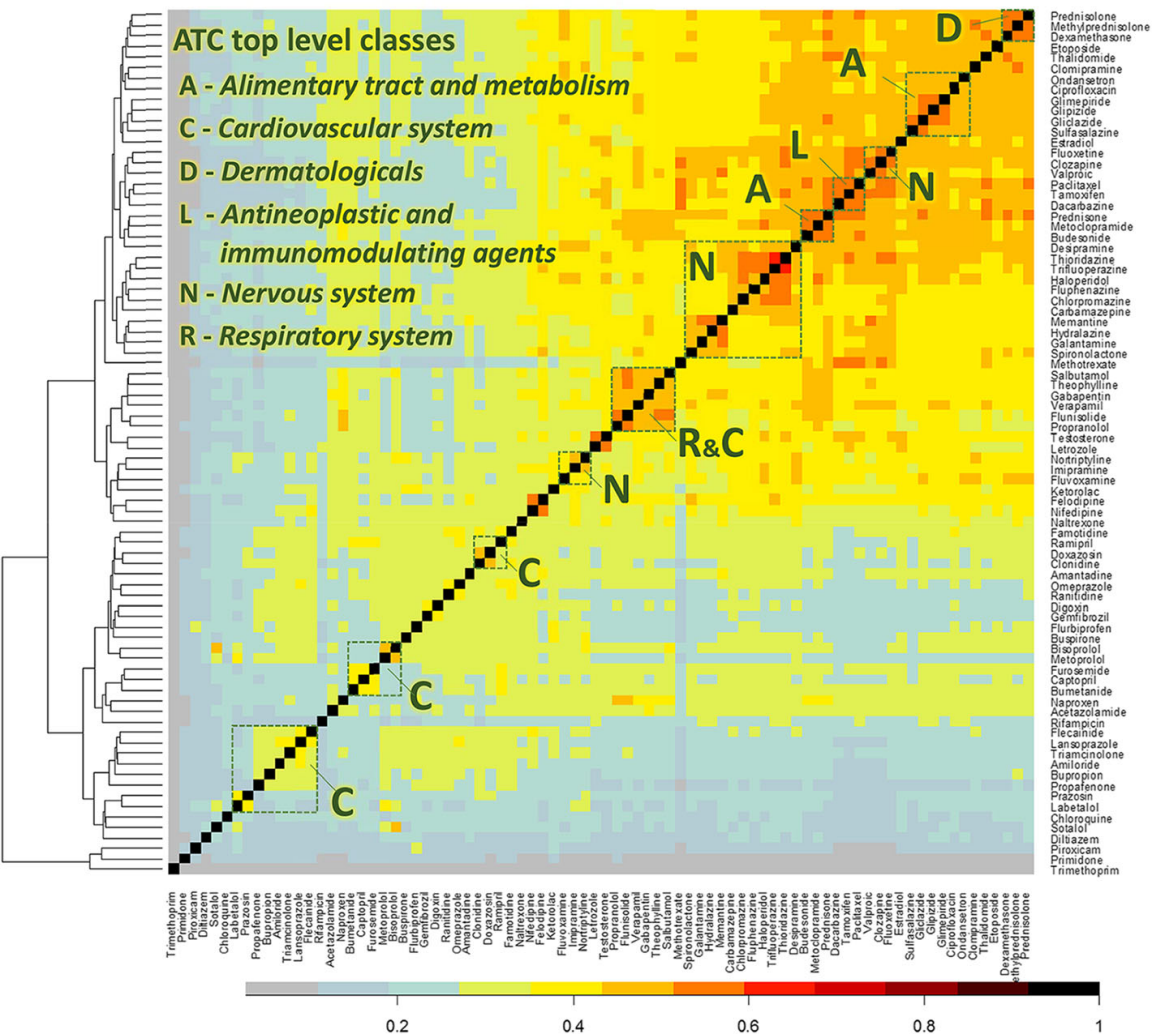
Heat map of similarity between drugs. The x-axis and y-axis indicate the same 82 drugs. Each cell depicts the similarity between two drugs based on a similarity score of their drug-specific pathways (Gene-Sim) and corresponding color key. Green dotted squares display the anatomical, therapeutic and chemical (ATC) top-level classes

In addition, drug clusters generated based on the similarity between drug-signaling pathways suitably reflect the anatomical, therapeutic and chemical (ATC) classification system [19], in which drugs are categorized according to their therapeutic properties. We were able to find that some unexpected drugs are included in the clusters and that these drugs can be regarded as candidates for drug repositioning, common side-effects and even drug-drug interactions (Fig. 4 and Additional file 1).

### Predicting new drug indications

TRANSFORMER utilizes drug-signaling pathways derived from Drug Voyager to predict novel drug indications under the assumption that drugs which have functionally similar pathways could have similar therapeutic effects (Fig. 1c). The known indications of a certain drug were assigned to other drugs which show significant similarity to that drug. Three approaches were applied to measure the functional similarity between drug-signaling pathways: Gene-Sim, GO-Sim and KEGG-Sim (Methods section).

**Fig. 5:**
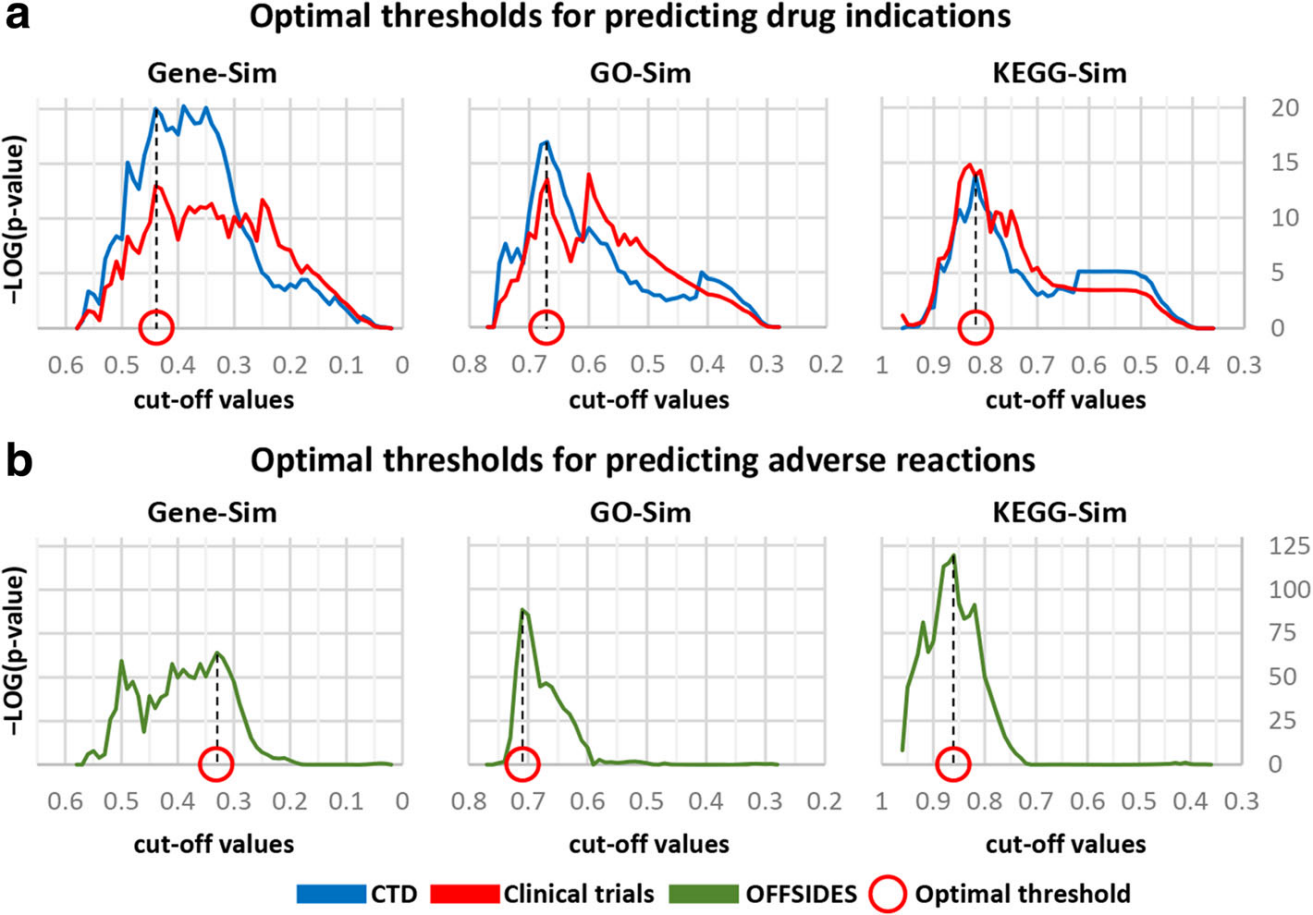
Selecting optimal thresholds for predictions. Selection of optimal thresholds for predicting drug indications (**a**) or adverse reactions (**b**) by considering the lowest enrichment P-value

To select the optimal cutoff for each similarity, we compare drug-disease pairs derived from each cutoff with those of the latest and most reliable repositories: 1) clinical trials [20], and 2) the Comparative Toxicogenomics Database (CTD) chemical-disease associations [18]. In Fig. 5, distinct cutoff values for which the output drug-disease pairs were strongly supported in both registries were identified for each drug-similarity measurement. By considering the most significant cutoff values, we obtained three optimal thresholds for three similarity measures, and they yielded three sets of drug-disease pairs. In a conservative manner, we only chose the intersections between the three sets. Consequently, we acquired 1,816 drug indications (Additional file 2). They were also significantly enriched in clinical trials (one-tailed Fisher’s exact *P* = 5.09E-23, odds ratio = 3.7` and the CTD (*P* = 1.30E-13, odds ratio = 4.3).

Furthermore, we evaluated our predictions using PubChem bioassays for cancers and compared these results to the prediction results obtained from Oh et al. [21] and Gottlieb et al. [6]. We queried the predicted drugs for each type of cancer and then acquired active or non-active records for each drug-cancer pair. In total, 345 drug-cancer pairs were aggregated. Using these pairs, we computed the F1 scores of the predictions identified by each method (Table 1). Our prediction shows better performance (F1 = 0.43) than the other methods in terms of its ability to predict active compounds for cancers.

**Table 1:** Evaluation of methods on PubChem bioassays for cancers. Performance of prediction results on cancer bioassays were displayed

Methods	F1 score	Recall	Precision	Odds ratio
TRANSFORMER	0.42	0.56	0.34	2.98
Gottlieb et al.	0.29	0.24	0.36	2.35
Oh et al.	0.16	0.13	0.19	0.67

### Predicting side effects of drugs

In addition to predicting drug indications, the same strategy was applied to infer drug-related side effects. Using the pathway-based similarities of drugs, the known side effects of a particular drug were assigned to other similar drugs. If the assigned side effects were in the known set, they were discarded. We applied three drug-drug-similarity measures (see Methods) and obtained a set of drug and side-effect pairs for each threshold and for each similarity measure. The three sets were selected using the optimal threshold for each similarity measure (Fig. 5b). Finally, the drug and side-effect associations that were included in all three sets were proposed as new side effects for existing drugs. In total, 11,152 predictions spanning 39 drugs and 1,598 side effects were determined in this manner (Additional file 3).

For validation purposes, drug-event associations were gathered from the OFFSIDES database [22], in which associations were statistically induced from the FDA Adverse Event Reporting System. The OFFSIDES database contains 438,801 off-label side effects for 1,332 drugs which are different from those in the SIDER database [23] (less than 5% overlap). Our predictions of drug side effects were significantly enriched in OFFSIDES (one-tailed Fisher’s exact *P* = 2.59E-95, odds ratio = 2.2).

Additionally, an adverse reaction database, Canada’s MedEffect, was used for further evaluation. In order to reduce confounding effects, we use only high-confidence reports that include exactly one drug which is suspected as the primary reason for an adverse reaction. Out of 30,664 drug-reaction pairs, 1,982 pairs involve drugs and side effects that belong to our data set (34 drugs and 529 side effects). Our predictions, which consist of 34 drugs and 529 side effects, significantly overlap with MedEffect (one-tailed Fisher’s exact P = 4.79E-09, odds ratio = 1.6).

## Discussion

In this study, we propose a computational platform to extract drug-signaling pathways for different drugs and its application to predictions of drug indications and side effects. By integrating genetic and phenotypic resources reflecting drug responses, we can formalize drug actions into drug-signaling pathways. Similarity between the drug-signaling pathways successfully led to meaningful candidates for drug indications and side effects.

To validate the computed drug-signaling pathways, we utilize the most comprehensive and reliable drug action pathway databases reflecting current knowledge of drug action. Despite considerable effort to accumulate drug action pathways, the curated pathways are limited to cover all of the computed pathways. Out of the 82 computed pathways, 30% of them are validated by SMPDB and only three drugs are validated by PharmGKB. The low coverages of known pathway databases might limit the applicability of this study. However, the further validation using text mining on literature was conducted for all 82 pathways and the statistical significance of 81 pathways was observed. Given the incompleteness of the curated pathways, a literature mining may reinforce the validation by covering the rest.

Also, we note that there can be a limitation when our method is applied to new compounds or drugs whose seed genes are not known because genes related to drug responses and phenotypes are required to construct the drug-signaling pathways. But the rapidly expanding databases and systematic prediction pipelines to secure drug-relevant genes may complement the restriction [13, 24–27].

Although our approach successfully obtains meaningful candidates for new drug uses and possible side effects, it is still limited with regard to its ability to offer insight into new mechanisms of drug actions. Here, we undertake a pathway analysis of the inferring mechanisms of unexpected drug actions based on drug-signaling pathways. To explore specific drug actions which are assumed to be responsible for new indications, we focus on the commonalities between drug-signaling pathways for both drugs, one a known treatment for a certain disease and the other a predicted one. Among the predicted drug indications, we highlight the following three indications as case studies: 1) haloperidol for Alzheimer’s disease, 2) propranolol for asthma and 3) thalidomide for prostate cancer. Also, we suggest a repositioning candidate for breast and prostate cancer, 4) fluphenazie, which is worth conducting further research on its feasibility of therapeutic application. Haloperidol is an antipsychotic agent which is used as a treatment for schizophrenia. The prediction of its use for Alzheimer’s disease originated from the similarity between haloperidol and valproic acid, which is a promising agent to combat Alzheimer’s disease [28] (Gene-Sim = 0.44, GO-Sim = 0.71, and KEGG-Sim = 0.87). The drug-signaling pathway of haloperidol has a large degree of overlap with that of valproic acid. Additional file 4: Figure S1 shows the shared portion. Genes in the shared part are annotated with the “Jak-STAT signaling pathway,” the “ErbB signaling pathway,” and the “vascular smooth muscle contraction pathway” in the KEGG pathway database using the DAVID tool (*P*-value < 0.05). These pathways have been studied and reported to be significantly linked to Alzheimer’s disease, as explained below. Dysregulation of the Jak-STAT signaling pathway is mainly associated with brain inflammation processes and neuronal/glial survival in the mature central nervous system (CNS). It is also involved in most brain disorders, including Alzheimer’s disease [29]. Seven genes, JAK1, JAK3, GRB2, IL2, PIK3R1, SOS1, and MYC, as shown in Additional file 4: Figure S1 are involved in the Jak-STAT signaling pathway. Aberration of ErbB signaling pathway have been deciphered as key regulatory entity in human diseases. Especially, deficiency of ErbB signaling is related to the development of neurodegenerative disorders, including Alzheimer’s disease and multiple sclerosis. [30]. Six genes, Gab1, GRB2, PIK3R1, SOS1, MYC, and Src, as shown in Additional file 4: Figure S1 are involved in the ErbB signaling pathway. It has been shown that the endophenotype-associated pathways of Alzheimer’s disease include vascular smooth muscle contraction, which was previously implicated in the biology of Alzheimer’s disease [31, 32]. Four genes, CYP4A11, GNA11, MYH11, and NPR1 in Additional file 4: Figure S1, are involved in vascular smooth muscle contraction.

Propranolol, a beta-adrenergic antagonist used predominantly for hypertension, was predicted to be efficient in the treatment of asthma. In our method, a basis for this prediction was two asthma medications, flunisolide and theophylline, which have similar drug-signaling pathways to that of propranolol. We found that drug-signaling pathways for these three drugs have large portions in common. Those shared pathway elements are shown in Additional file 5: Figure S2, and the genes are annotated with “MAPK signaling pathway”, “Neurotrophin signaling pathway”, and “VEGF signaling pathway” in the KEGG pathway database using DAVID tools (P-value < 0.05). Those pathways have been demonstrated to have a significant relationship with asthma in the following studies. Asthmatic patients demonstrated increased immunostaining for phospho (p)-ERK1/2, p-p38α/β/γ (p-p38), and pJNK1/2/3 (pJNK) [33], which are important members of the MAPK signaling pathway, and are also shown in Additional file 5: Figure S2. Neurotrophins and their receptors are expressed in lung components, and the neurotrophin signaling pathway may be important in normal lung development, developmental lung disease, and allergy and inflammation (e.g., rhinitis, asthma) [34]. Eight genes including MAPK3, MAPK1, MAPK14, MAPK8, MAP2K7, MAP3K1, RPS6KA6, and RAF1 in Additional file 5: Figure S2 are involved in the neurotrophin signaling pathway. Elevated VEGF levels have been observed in patients with asthma [35]. Furthermore, it has also been suggested [36] that VEGF excess can contribute to the pathogenesis of Th2 inflammatory disorders such as asthma. Six genes including MAPK3, MAPK1, MAPK14, PLA2G1B, RAF1, and SRC in Additional file 4: Figure S2 are involved in the VEGF signaling pathway.

We predict that thalidomide can be used for prostate cancer. Thalidomide, an immunosuppressive agent, was once withdrawn from the market because of its teratogenic effects and has been reintroduced and used for immunological diseases. Thalidomide’s drug-signaling pathway substantially overlaps with a drug-signaling pathway for estradiol which has a therapeutic effect on prostate cancer [37]. Additional file 6: Figure S3 shows the overlapping pathway. The genes in Additional file 6: Figure S3 are annotated with “TCA cycle”, “pyruvate metabolism”, and “Wnt signaling pathway” in the KEGG pathway database, using DAVID tools (P-value < 0.05). Those pathways have been reported and observed to have a significant relationship with prostate cancer, as detailed below. It has been reported [38] that there are significant changes in citrate-related metabolism and transport in prostate cancer. The tricarboxylic acid (TCA) cycle is also linked to the excess production of reactive oxygen species (ROS). As excess ROS causes damage to DNA, RNA and proteins, it leads to oxidative stress, including metabolic alteration and mitochondrial dysfunction which accelerate tumorigenesis in prostate cancer. Five genes including DLAT, DLD, PC, PDHA2, and PDHB were involved in the TCA cycle. Those five genes were also found in pyruvate metabolism, and pyruvate is used for metabolic imaging of prostate cancer [39]. Disruption or dysregulation of the Wnt signaling pathway can lead to the development of many tumors including prostate cancer [40]. The Wnt/β-catenin pathway may regulate prostate tumor cells’ invasive behavior, mediating cell proliferation and epithelial-mesenchymal trans-differentiation [41]. CTBP1, JUN, and MYC in Additional file 6: Figure S3 are downstream genes in the Wnt signaling pathway.

Fluphenazine is one of phenothiazine antipyscotics, which is categorized as dopamine receptor antagonists and calmodulin inhibitors. TRANSFORMER predicted its new indication for breast and prostate cancer. It was found that a drug-signaling pathway of this agent shares substantial members with that of estradiol, which has been reported to be used for treatment of breast and prostate cancer. To identify functional commonality, GO annotation analysis was performed on the shared part of their pathways. Table 2 shows the top 10 GO enrichment results. Among them, we focus on cyclin-dependent kinase (CDK) activity and G-protein coupled receptor (GPCR) signaling pathway. It is clear that CDK family is principal to several signaling pathways regulating transcription and cell-cycle progression. In year 2013, FDA approved CDK4 and CDK6 inhibitors for breast cancer as breakthrough therapies [42, 43]. We found that the shared part of fluphenazine and estradiol drug-signaling pathways involves several CDK4/6 inhibitor genes, including CDKN2A, CDKN1B and CDKN1A. The other interesting CDK protein in the shared part is CDK5, which has multiple roles in some tissues with relevance to cancers [44]. GPCR controls key physiological functions [45]. It is reviewed that GPCRs can be crucial players in tumor growth and metastasis [46]. Especially, CHRM3 gene which is annotated to GO term of GPCR signaling pathway was identified in the shared part. In the recent study, it has been found that autocrine activation of CHRM3 promotes prostate cancer growth [47]. These findings could support the potential indication of fluphenazine for breast cancer and prostate cancer.

**Table 2:** The top ten Gene Ontology terms enriched. GO annotation analysis was performed on the shared members derived from the drug-signaling pathways of fluphenazine and estradiol

GO term	Description	P-value	FDR q-value
GO:1904029	regulation of cyclin-dependent protein kinase activity	1.11E-09	9.24E-06
GO:0000079	regulation of cyclin-dependent protein serine/threonine kinase activity	1.11E-09	4.62E-06
GO:0051726	regulation of cell cycle	1.28E-09	3.54E-06
GO:0051301	cell division	1.37E-09	2.83E-06
GO:0051290	protein heterotetramerization	4.61E-09	7.64E-06
GO:0034723	DNA replication-dependent nucleosome organization	1.55E-08	2.14E-05
GO:0006335	DNA replication-dependent nucleosome assembly	1.55E-08	1.83E-05
GO:0006334	nucleosome assembly	1.58E-08	1.63E-05
GO:0051262	protein tetramerization	4.87E-08	4.48E-05
GO:0007186	G-protein coupled receptor signaling pathway	5.95E-08	4.94E-05

Additionally, we discuss a few predicted adverse drug reactions based on published studies and the US FDA Online Label Repository. Although the FDA drug label is biased toward the number of occurrences of adverse reactions that are observed and reported in clinical trials or post-marketing surveillance, it is one of the clearest options for comparison to the predicted results. We highlight the predicted adverse reactions of the three drugs paclitaxel for bradycardia and tachycardia, valproic acid for delirium and neutropenia, and tamoxifen for hypothyroidism. Paclitaxel, a tubulin modulator used in chemotherapy, was predicted to induce bradycardia and tachycardia. In one phase 2 study, 29% of the 45 patients who were treated with paclitaxel developed bradycardia [48]. In a subsequent large-cohort study, a rate of 0.1% of cardiac toxicity was reported, and most of these cases were asymptomatic bradycardia. In rare cases, atrial and ventricular tachycardia were described [49]. Another study assessing cardiac disturbances in one hundred African-American patients treated with paclitaxel found that 26% of patients experience sinus tachycardia [50].

Valproic acid, which is administered predominantly in epilepsy and psychiatric disorders, was suspected to cause delirium in our prediction results. Delirium had been reported in three studies in which valproic acid levels in plasma were within therapeutic ranges [51–53]. Although delirium is excluded from the adverse reactions described in the FDA drug labeling for valproic acid, the most common form of delirium, hyperactivity, is included in the labeling. In addition to delirium, neutropenia appeared in our predictions for valproic acid. Likewise, neutropenia following valproic acid exposure was also observed in other studies [54–56]. The FDA drug label does not specify neutropenia as an adverse reaction for valproic acid, but leukopenia is labeled. As the most common subtype of leukopenia is neutropenia, this label corroborates that our prediction is supported in the current pharmacovigilance system.

Tamoxifen, a selective estrogen receptor modulator (SERM) used predominantly for the treatment of breast cancer, was inferred to be a possible agent responsible for hypothyroidism by our method. The FDA drug label does not include warnings for drug-induced hypothyroidism. However, particularly in postmenopausal breast cancer patients, significant alterations in thyroid function tests were observed during treatment with tamoxifen [57]. Specifically, the previous study reports that tamoxifen treatment significantly elevates plasma levels of thyroid-stimulating hormone (TSH) and significantly suppresses free triiodothyronine (FT3) and free thyroxine (FT4). As the diagnosis of hypothyroidism is confirmed by an elevated TSH level and a low FT4 level, we can suspect that tamoxifen may have an effect in inducing hypothyroidism. In a more recent study, the authors raise caution related to treating thyroid dysfunction in women who are taking SERMs such as tamoxifen [58].

We further note that it was difficult to find literature evidence of predictions of severe adverse reactions such as cardiac arrest and coma, as we found that the more the relative severity of the adverse reactions increases, the fewer reports there are available (Additional file 7: Figure S4).

## Conclusions

Verifiable hypotheses about unintended drug actions are implicitly inherent in emerging data relevant to drug responses. Our study shows how we can utilize these data to computationally represent drug-signaling pathways. Furthermore, computational analysis of drug-signaling pathways can provide more precise predictions of drug-repositioning candidates and adverse reactions as well as the mechanisms of unintended actions.

## Methods

### Datasets

#### Drug-specific genes

The five types of genes relevant to drug responses and phenotypes were obtained and assembled for each drug from the following references. First, drug target proteins were obtained from DrugBank [24] and mapped to corresponding genes. Second, the pharmacogenomic variants associated with the drug responses were extracted from PharmGKB [13] and mapped to the assigned genes. Third, small-molecule gene expression profiles were gathered from Connectivity Map [59]. For each drug treatment, a differentially expressed gene set relative to a vehicle-treated control was selected from the MCF7 cell lines. The gene set consisted of probe sets ranked in the top 50 and bottom 50. Fourth, disease genes were extracted from the Online Mendelian Inheritance in Man (OMIM) database [25] and known drug-disease associations were collected from Gottlieb et al. [6] Disease genes were assigned to drugs which are known to be associated with the diseases. Fifth, genes related to side effects were collected from Gottlieb and Altman [9] and known associations between drugs and side effects were extracted from the SIDER database [23] (July 2015). Side-effect genes were assigned to drugs if these drugs are known to be associated with the side effect. In brief, 82 drugs with at least one gene of each gene type were used out of the entire group of FDA-approved drugs selected from DrugBank. The average number of drug-specific genes for each drug was 165 ± 43.

#### Biological networks

Curated pathways were extracted from the Pathway Interactions Database [60], BioCarta [61], and Reactome [62]. The union of the pathway interactions includes approximately 50,000 directed interactions and more than 14,000 biomolecules consisting of RNAs, proteins, compounds, and complexes of these. Protein-protein interactions (PPIs) were aggregated from multiple sources, including BioGrid [63] (version 3.3.124), the Database of Interacting Proteins [64] (May 2015), IntAct [65] (May 2015), and the Molecular Interaction Database [66] (May 2015). The integration of the PPIs led to more than 400,000 interactions.

#### Known drug indications

In order to predict new drug indications on the basis of current use, known drug indications were obtained from the gold standard [6]. Out of 1,933 indications spanning 593 drugs and 313 diseases, we obtained 414 indications as known drug indications for 82 experimental drugs. These encompass 155 diseases.

#### Known drug side effects

To predict new adverse reactions, 97,755 drug and side-effect pairs were downloaded from the SIDER database (July 2015). Among them, 15,630 pairs that contain drugs for which it is possible to generate a drug-signaling pathway were used as the known side effects of drugs. For validation purposes, drug-event associations were obtained from the OFFSIDES database [22]. These associations were statistically detected from the FDA Adverse Event Reporting System. The OFFSIDES database includes 438,801 off-label side effect events for 1,332 drugs. Among them, 10,334 drug-event associations were preprocessed for 82 drugs.

##### A platform for building a drug-signaling pathway

Drug Voyager was designed to construct a drug-signaling pathway for each drug (Fig. 1a). In order to render the drug response pathways realistically, three conceptual levels were used, and these were termed “initiation,” “perturbation,” and “destination.” Each level had specific element genes relevant to the drug response or phenotype. There are five types of element genes: drug target genes (TG), pharmacogenomic variant gene (VG), differentially expressed genes (DEG), disease genes (DisG), and side-effect genes (SEG). Based on the assumption that drug targets initiate a signaling cascade that ultimately affects the disease phenotype, the TGs were regarded as components of the initiation level. The VGs and DEGs were assigned to the perturbation level. Then, the DisGs and SEGs were added to the destination level. Drug Voyager reconstructs level-to-level pathways using the construction method described below (i.e., from the initiation level to the perturbation level, and from the perturbation level to the destination level). By combining level-to-level pathways, a consequent drug-signaling pathway was generated for each drug.

##### Constructing a pathway from the starting point to the end point

A pathway was constructed by connecting the given start points and end points based on the interactions determined from biological networks (Fig. 1b). These networks consist of the manually curated pathway interactions, including signaling pathways, regulatory pathways, and metabolic pathways. Moreover, the pathways were established to propagate signals between diverse biomolecules such as genes, chemical compounds, and complexes, rather than only the signals between genes. Out of all paths from the start points to the end points, the shortest paths were generated by traversing the curated pathway interactions in the given directions. Among them, the particular paths in which the last interaction is the regulation of transcription or translocation were considered as the resulting pathways. If no paths were connected, the curated PPIs were allowed to compose the front of the paths up to three interactions, linking the start points to known pathway components.

##### Predicting drug-repositioning candidates and new adverse reactions

We devised a novel method, TRANSFORMER, to translate the functional features of a drug-signaling pathway into a new medicine and its adverse reactions. The drug-signaling pathway constructed by Drug Voyager was applied to infer new drug indications, including therapeutic uses and adverse reactions (Fig. 1c). Given the assumption that some drugs which have functionally similar pathways could have similar therapeutic effects, three approaches were applied to measure functional similarity between drug-signaling pathways. These were the gene component similarity (Gene-Sim), gene ontology (GO) enrichment similarity (GO-Sim), and the Kyoto Encyclopedia of Genes and Genomes (KEGG) enrichment similarity (KEGG-Sim) approaches. For Gene-Sim, a binary vector was generated for a drug-signaling pathway to indicate gene membership of a corresponding pathway. For GO-Sim, the enrichment in the gene ontology for a biological process was observed to annotate each drug-signaling pathway, and a binary vector depicts the involvement of the biological process (*P*-value < 0.01, using DAVID [67]). In addition to GO-Sim, a snapshot of the pathway cross-talk was obtained based on the enrichment test. For KEGG-Sim, a binary vector indicating significantly enriched KEGG pathway membership was generated for each drug-signaling pathway (*P*-value < 0.01, using DAVID [67]). Sequentially, the Jaccard coefficient was used to calculate the degree of similarity between two binary vectors for each approach.

Based on the similarity measures and the given threshold for each similarity, known indications of a certain drug were assigned to other drugs which show significant levels of similarity to the original drug. If the assigned drug indication was found among the known indications, it was removed. As a result, the assigned drug indications are independent of the known indications. Only cases in which the newly assigned drug-disease pairs satisfied all three measurements were considered to be new predictions.

To determine the optimal threshold, newly assigned drug-disease pairs were statistically compared to valid drug-disease associations, for which clinical trials and the curated drug-disease relationship were used. The threshold resulting in the lowest P-value was selected for each similarity measure. To predict new adverse reactions, the same method described above was used. The known side effects of drugs were employed instead of the known indications. In order to determine the optimal threshold, we used OFFSIDES, an off-label side-effects database [21].

##### Statistics in enrichment test

To identify significant enrichment in the reference annotation, we used hypergeometric test. If a query contains valid entities (i.e. genes) from a total of t entities, for a given annotation (i.e. GO term), there are q entities within k and m entities within t associated with it, then the possibility that whether entities associated with this annotation is enriched among the queried entity list could be calculated by hypergeometric test,

$$ P\left( X= x> q\right)={\displaystyle \sum_{x= q}^m\frac{\left({}_x^m\right)\left({}_{k- x}^{t- m}\right)}{\left({}_k^t\right)}}. $$

## Additional files

Additional file 1: (1) Constructing a drug-specific pathway. **Figure S5.** Schematic illustration of component-specific pathway construction. (2) Prediction models based on drug similarity. (3) Drug clusters based on drug-signaling pathways. (4) PID molecule ID notation for **Figure S1**, **S2** and **S3.** **Figure S6.** PID molecule ID notation for **Figure S1.** **Figure S7.** PID molecule ID notation for **Figure S2.** **Figure S8.** PID molecule ID notation for **Figure S3.** (DOCX 874 kb)
Additional file 2: Predicted drug indications. The list of 1,816 novel drug indications spanning 47 drugs and 122 diseases. (XLSX 54 kb)
Additional file 3: Predicted side effects of drugs. The list of 11,152 novel side effects of drugs spanning 39 drugs and 1,598 side effects. (XLSX 179 kb)
Additional file 4: Figure S1. The overlap between drug-signaling pathways (haloperidol and valproic acid). The shared pathways derived from each drug-signaling pathway for haloperidol and valproic acid. (JPG 933 kb)
Additional file 5: Figure S2. The overlap between drug- signaling pathways (propranolol, flunisolide, and theophylline). The shared pathways derived from each drug-signaling pathway for propranolol, flunisolide, and theophylline. (JPG 1113 kb)
Additional file 6: Figure S3. The overlap between drug- signaling pathways (thalidomide and estradiol). The shared pathways derived from each drug-signaling pathway for thalidomide and estradiol. (JPG 973 kb)
Additional file 7: Figure S4. The relationship between severity and frequency of the adverse reactions. Each point of the scatter plot represents a side effect. X-axis shows its frequency in known drug-side effect pairs and Y-axis displays the relative severity. (JPG 481 kb)

## Abbreviations

API: Application program interface
ATC: The anatomical, therapeutic and chemical
CNS: Central nervous system
CPIC: Clinical Pharmacogenetics Implementation Consortium
CTD: Comparative Toxicogenomics Database
DEG: Differentially expressed genes
DisG: Disease genes
FDA: Food and Drug Administration
FT3: Free triiodothyronine
FT4: Free thyroxine
Gene-Sim: gene component similarity
GO: Gene ontology
GO-Sim: Gene ontology enrichment similarity
KEGG: Kyoto Encyclopedia of Genes and Genomes
KEGG-Sim: Kyoto Encyclopedia of Genes and Genomes enrichment similarity
NCBI: National Center for Biotechnology Information
OMIM: Online Mendelian Inheritance in Man
PPIs: Protein-protein interactions
ROS: Reactive oxygen species
SEG: Side-effect genes
SERM: Selective estrogen receptor modulator
SMPDB: Small Molecule Pathway Database
TCA: The tricarboxylic acid
TG: Drug target genes
TSH: Thyroid-stimulating hormone
VG: Pharmacogenomic variant genes
