# Supplementary material for: Drug voyager: a computational platform for exploring unintended drug action
Source: BMC Bioinformatics. 2017 Feb 28;18:131. doi: 10.1186/s12859-017-1558-3 (PMC5329936; doi:10.1186/s12859-017-1558-3)
Supplement: Additional file 1: — (1) Constructing a drug-specific pathway. Figure S5. Schematic illustration of component-specific pathway construction. (2) Prediction models based on drug similarity. (3) Drug clusters based on drug-signaling pathways. (4) PID molecule ID notation for Figure S1, S2 and S3. Figure S6. PID molecule ID notation for Figure S1. Figure S7. PID molecule ID notation for Figure S2. Figure S8. PID molecule ID notation for Figure S3. (DOCX 874 kb) [file 12859_2017_1558_MOESM1_ESM.docx]

Additional file for

Drug Voyager:

A Computational Platform for Exploring Unintended Drug Action

Min Oh^1^, Jaegyoon Ahn^2^, Taekeon Lee^3^, Giup Jang^3^, Chihyun Park^4^ and Youngmi Yoon^3,*^

^1^Department of Computer Science, Virginia Tech, Blacksburg, VA, USA

^2^Department of Computer Science & Engineering, Incheon National University, Incheon, Korea

^3^Department of Computer Engineering, Gachon University, Seongnam, Korea

^4^Biomedical HPC Technology Research Center, Korean Institute of Science and Technology Information, Daejeon, Korea

*To whom correspondence should be addressed.

E-mail: [ymyoon@gachon.ac.kr](mailto:ymyoon@gachon.ac.kr)

**Contents**

1. Constructing a drug-specific pathway

2. Prediction models based on drug similarity

3. Drug clusters based on drug-signaling pathways

4. PID molecule ID notation for Figure S1, S2 and S3.

1. **Constructing a drug-specific pathway**


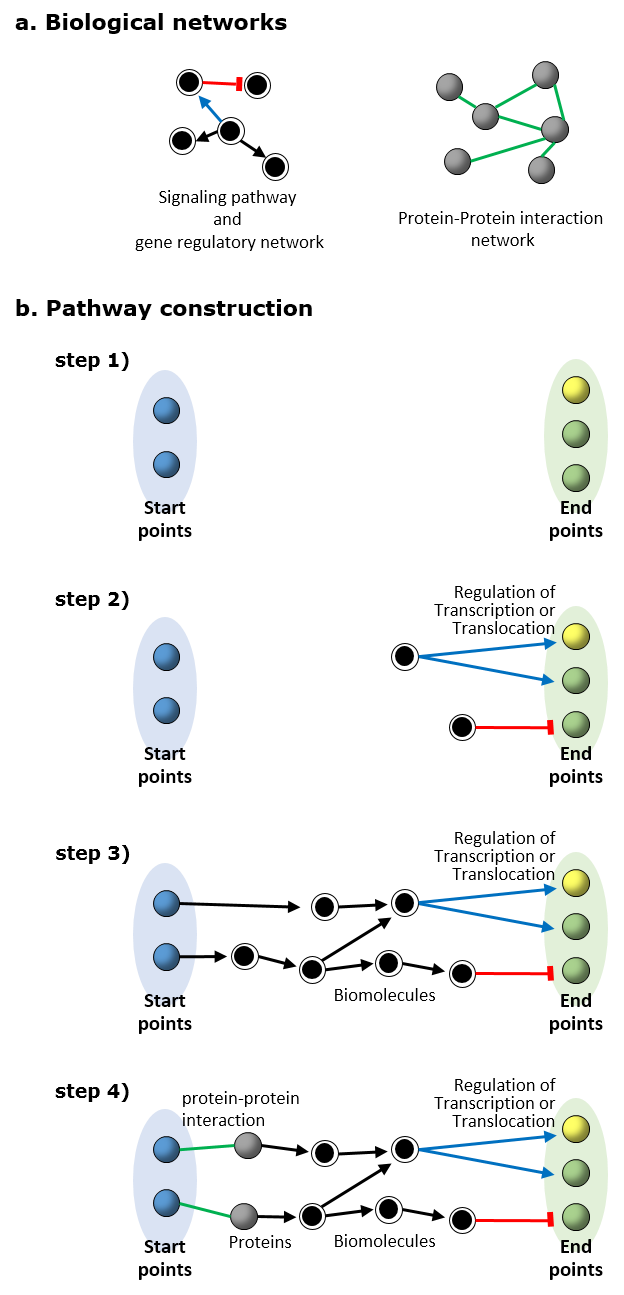


Figure S5. Schematic illustration of component-specific pathway construction

A component-oriented pathway was constructed based on input components and biological networks. We assume that the signaling cascade ultimately affects gene regulation. Given two set of components, one termed start points and the other end points, shortest signal propagations are captured as follows:

1. Check whether start points are available in biological networks and check whether end points are available in the gene regulatory network.
2. Find regulatory components that regulate the end points.
3. Find the shortest signaling paths from the start points to the regulatory components.
4. If failing to find any paths in 3), then find the shortest paths from the neighbors of the start points in protein-protein interactions to the regulatory components.

(e.g., Start points can be drug targets and end points can be pharmacogenomics variants.)

Despite the best efforts to curate a signaling pathway network and gene regulatory networks, these networks remain incomplete. Hence, many paths from drug targets to drug-response-related genes are lost. In order to overcome this handicap, we used the curated PPI to connect start points to the signaling network. We define the neighbors of start points as components that are linked to the start points by up to three interactions in the protein-protein interactions.

1. **prediction models based on drug similarity**

Various drug-similarity measures have been used to identify new drug indications, and some of them have shown success in predictions of drug indications. We selected three traditional similarity measurements that consistently show the best prediction performances.1,2

1. Chemical similarity: The chemical similarity between two drugs was calculated based on their fingerprints downloaded from DrugBank, and using Open Babel. 3
2. Drug target similarity: We downloaded drug target proteins from DrugBank. We calculated the proportion of common drug targets using the Jaccard index.
3. Side-effect similarity: Side effects of drugs were obtained from the SIDER database. The side-effects similarities between drugs were measured using the Jaccard index.

We use the maximum value of the similarity measure.

## Drug clusters based on drug-signaling pathways

The basic assumption of TRANSFORMER is that some drugs that have similar drug-signaling pathways could have similar therapeutic effects. In order to apply the assumption, it is critical to test that drug clusters generated based on the similarity between drug-signaling pathways reflect the current drug classification system in which drugs are categorized according to their therapeutic properties. In Figure 3, a heat map depicts drug clusters based on Gene-Sim, which is a measure of similarity between drug-signaling pathways (see Methods). The heat map is overlaid with top level classes of the Anatomical, Therapeutic and Chemical (ATC) classification system4, and indicates that the drug clusters coincide with the ATC classification. For instance, the “D cluster” in Figure 3 includes three dermatological drugs, prednisolone, methylprednisolone, and dexamethasone, which are corticosteroids.

The drug clusters usually reflect the ATC classification. However, we were able to find that some unexpected drugs are included in the clusters. In this study, the unexpected drugs were regarded as candidates for drug repositioning or common side-effects. However, we note that the unexpected drugs can be considered candidates for drug interaction with other drugs in the clusters. For example, there is an “A cluster” in Figure 3 (the big one of two A clusters) which consists of six drugs, ondansetron, ciprofloxacin, glimepiride, glipizide, gliclazide, and sulfasalazine (Figure 3). With the exception of one drug, ciprofloxacin, the other five drugs are classified as ATC top level class of “A”, which indicates the alimentary tract and metabolism group. Among the five, three drugs, glimepiride, glipizide, and gliclazide are diabetes drugs. In the literature, we found that ciprofloxacin, an anti-infective agent, has been reported to interact with oral medicine for diabetes, including glimepiride, glipizide, and gliclazide.5-7

## PID molecule ID notation for Figure S1, S2 and S3.


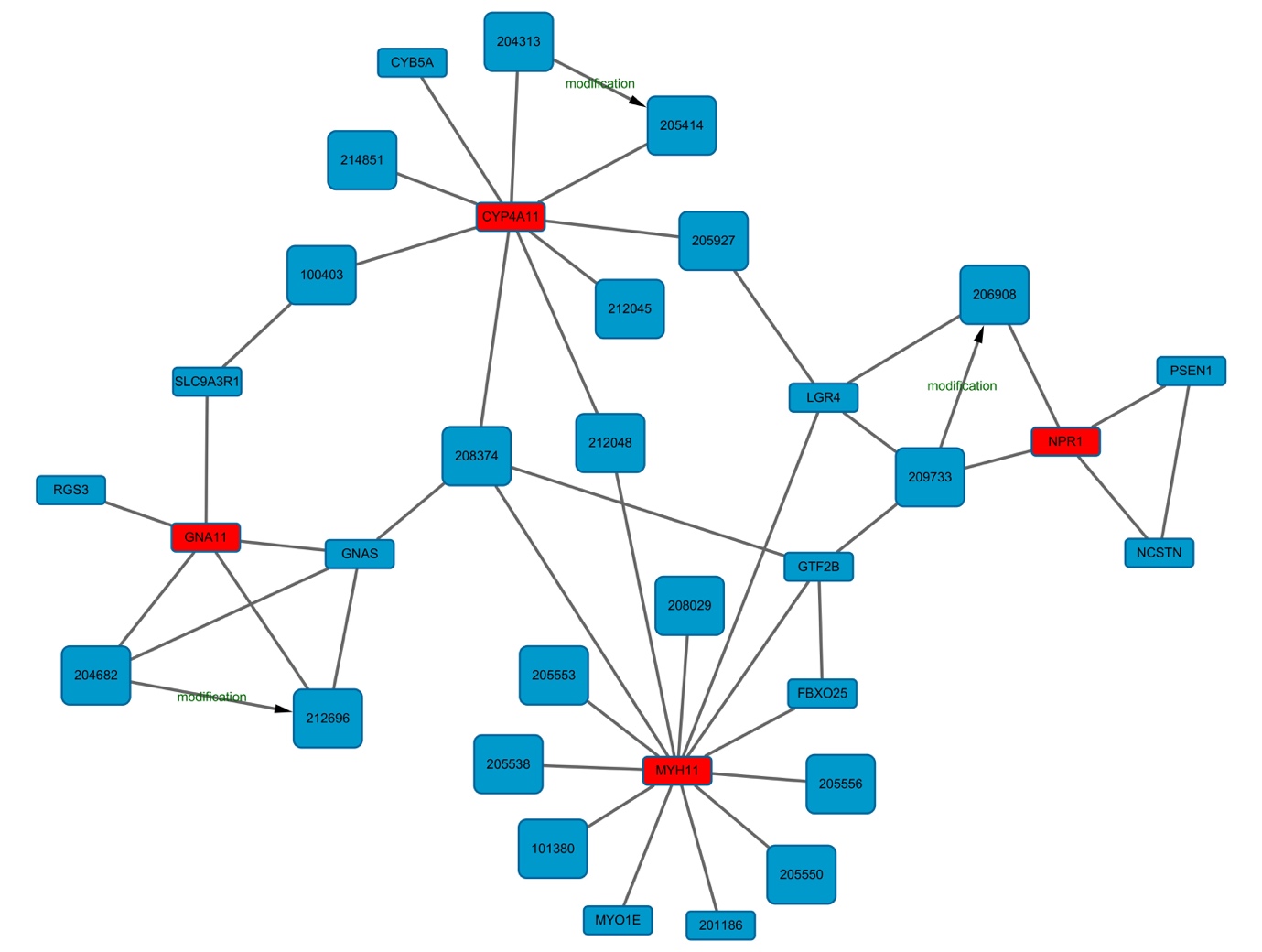


Figure S6. PID molecule ID notation for Figure S1.


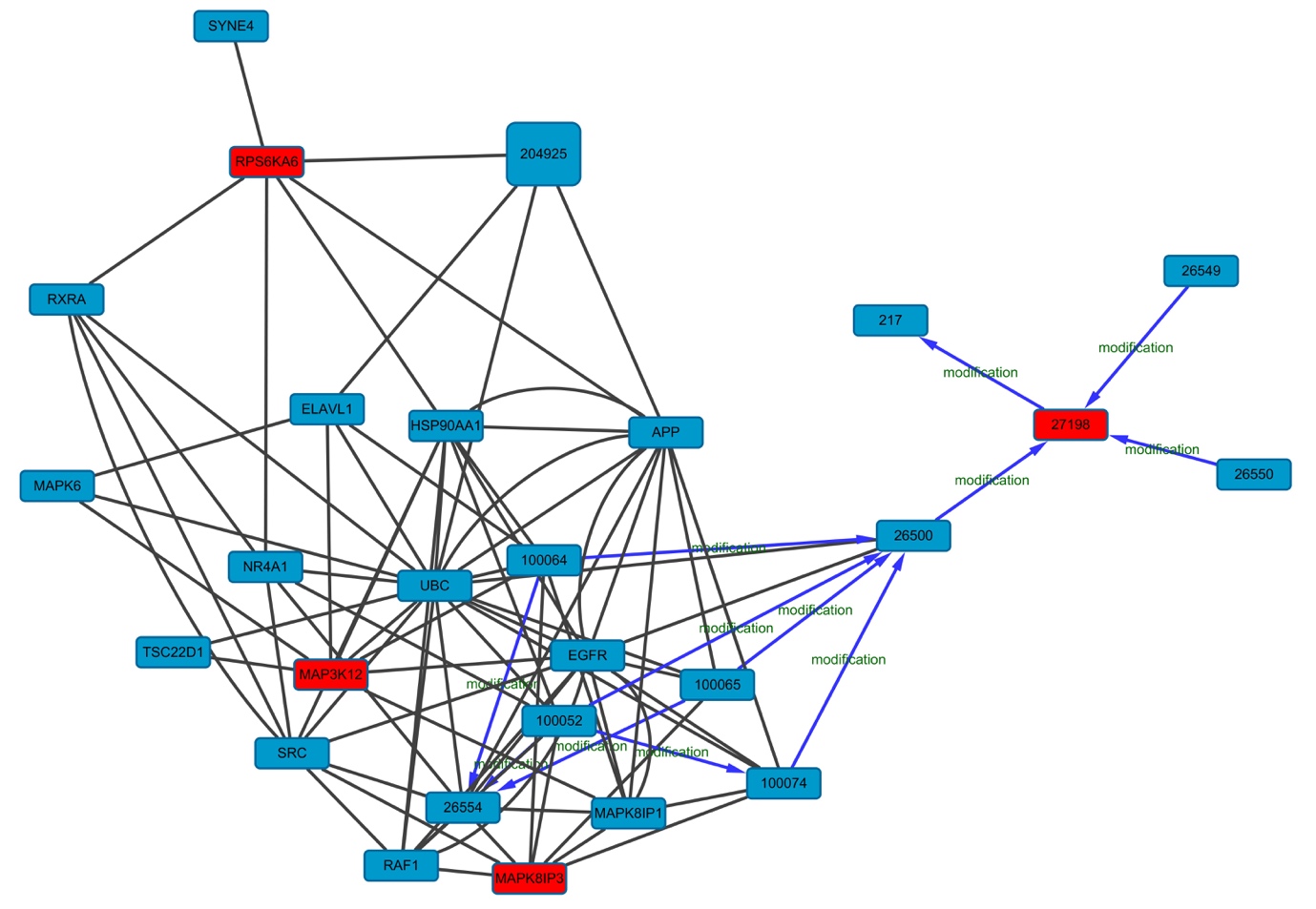


Figure S7. PID molecule ID notation for Figure S2.


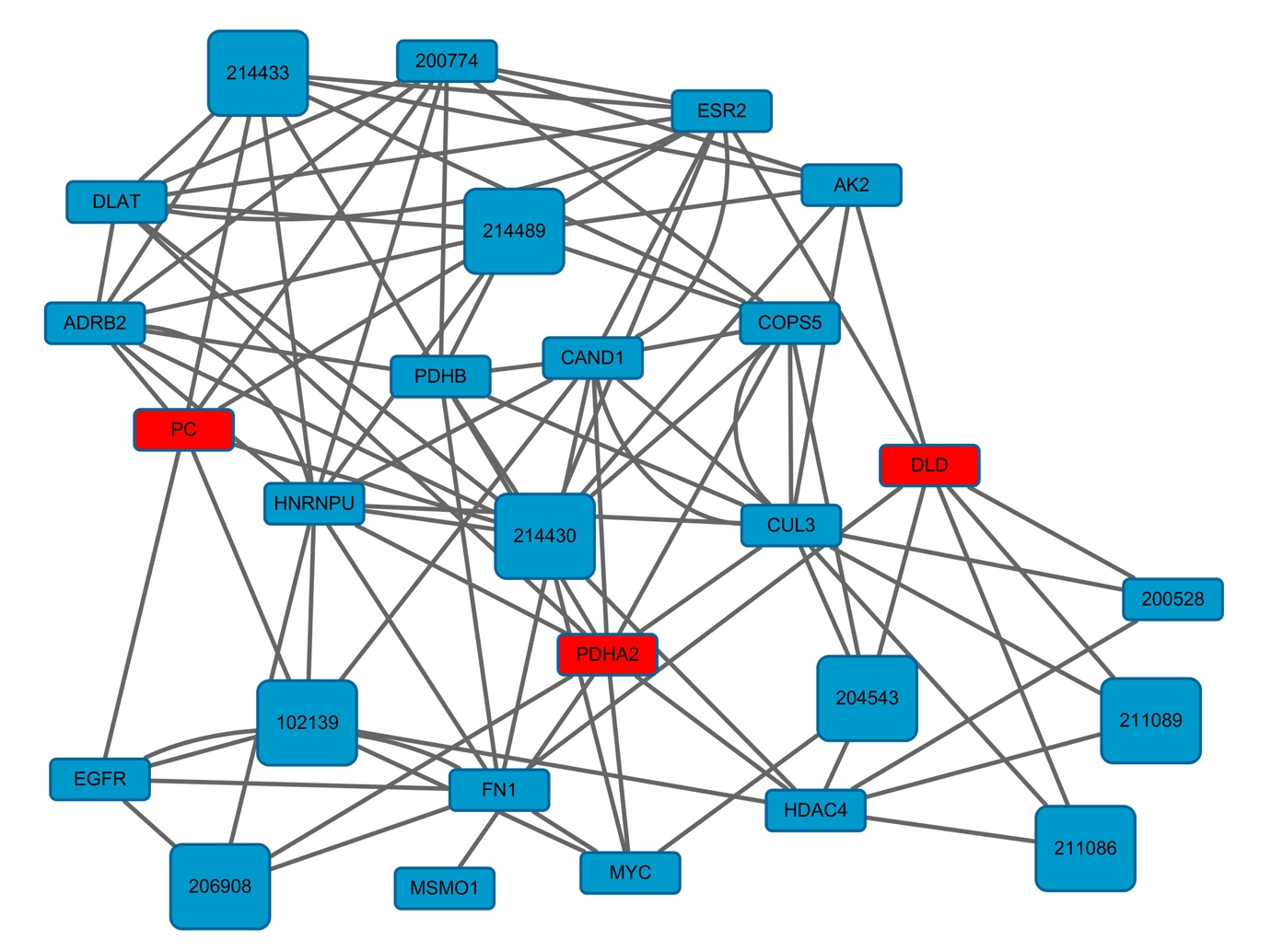


Figure S8. PID molecule ID notation for Figure S3.

**References**

1. Gottlieb, A., Stein, G. Y., Ruppin, E., & Sharan, R. PREDICT: a method for inferring novel drug indications with application to personalized medicine. Mol Syst Biol, 7, 496 (2011).

2. Guney, E., Menche, J., Vidal, M., & Barábasi, A. L. Network-based in silico drug efficacy screening. Nat Commun, 7 (2016).

3. O'Boyle, N. M., Banck, M., James, C. A., Morley, C., Vandermeersch, T., & Hutchison, G. R. Open Babel: An open chemical toolbox. J Cheminform, 3, 1 (2011).

4. Skrbo, A., Begović, B., & Skrbo, S. Classification of drugs using the ATC system (Anatomic, Therapeutic, Chemical Classification) and the latest changes. Med Arh, 58, 138 (2004).

5. Covvey, J., & Lewis, D. Glimepiride-induced hypoglycemia with ciprofloxacin, metronidazole, and acute kidney injury. Hosp Pharm, 45, 934-938 (2010).

6. Schelleman, H., Bilker, W. B., Brensinger, C. M., Wan, F., & Hennessy, S. Anti‐Infectives and the Risk of Severe Hypoglycemia in Users of Glipizide or Glyburide. Clin Pharmacol Ther, 88, 214-222 (2010).

7. Mayo Clinic, http://www.mayoclinic.org/
