# Supplementary figures and images for: Drug voyager: a computational platform for exploring unintended drug action
Source: BMC Bioinformatics. 2017 Feb 28;18:131. doi: 10.1186/s12859-017-1558-3 (PMC5329936; doi:10.1186/s12859-017-1558-3)

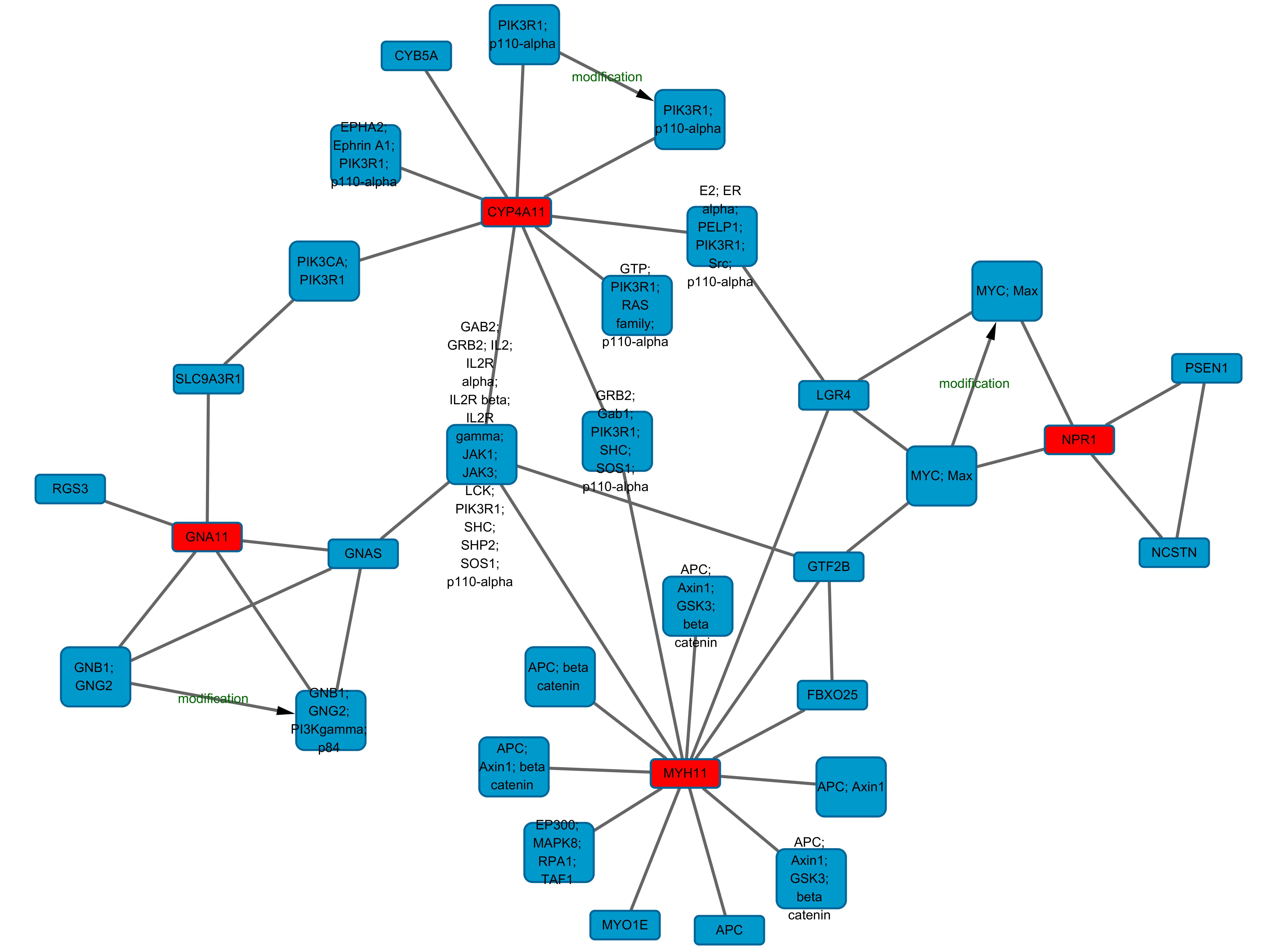

Supplement: Additional file 4: Figure S1. — The overlap between drug-signaling pathways (haloperidol and valproic acid). The shared pathways derived from each drug-signaling pathway for haloperidol and valproic acid. (JPG 933 kb) [file 12859_2017_1558_MOESM4_ESM.jpg]

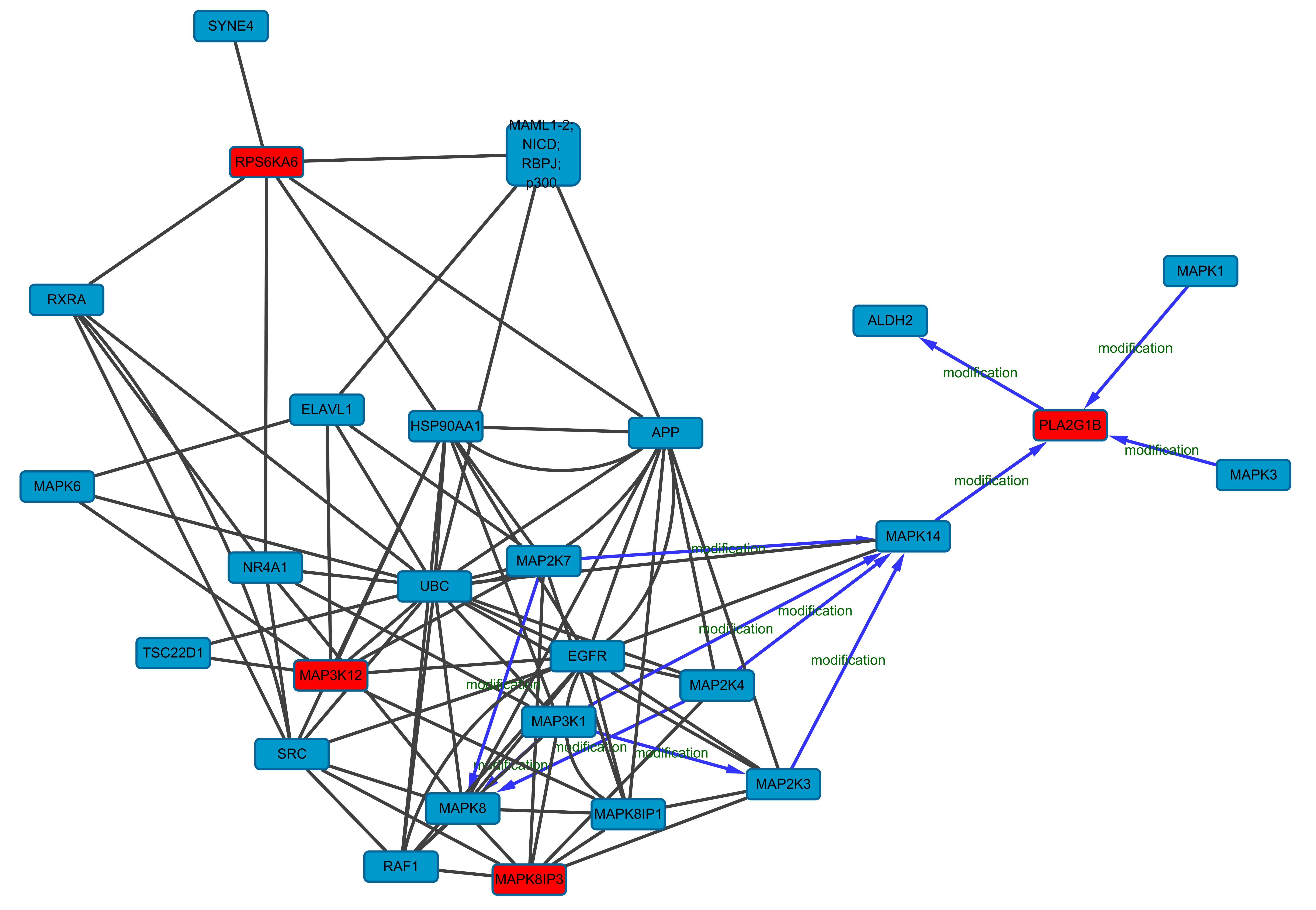

Supplement: Additional file 5: Figure S2. — The overlap between drug- signaling pathways (propranolol, flunisolide, and theophylline). The shared pathways derived from each drug-signaling pathway for propranolol, flunisolide, and theophylline. (JPG 1113 kb) [file 12859_2017_1558_MOESM5_ESM.jpg]

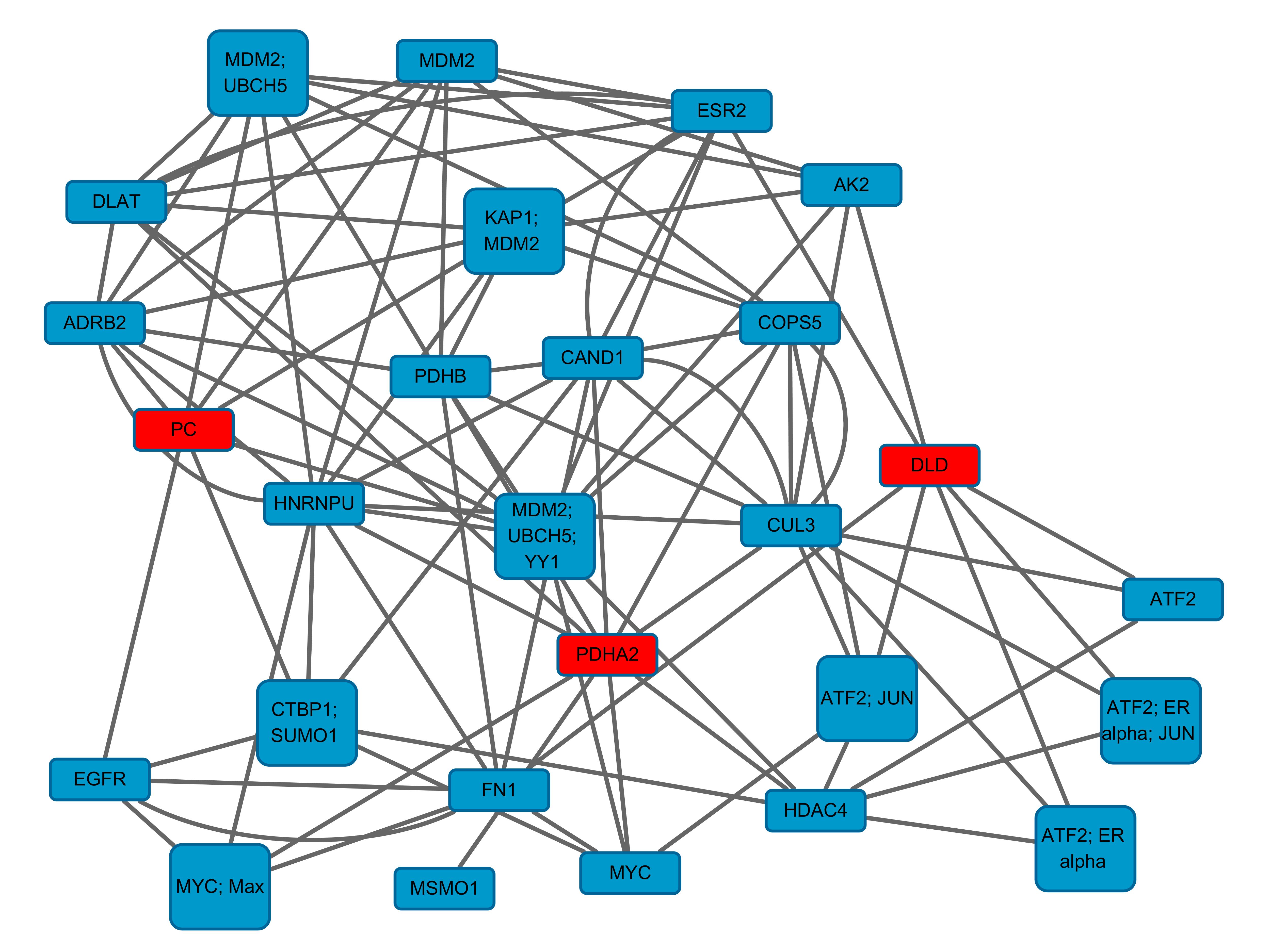

Supplement: Additional file 6: Figure S3. — The overlap between drug- signaling pathways (thalidomide and estradiol). The shared pathways derived from each drug-signaling pathway for thalidomide and estradiol. (JPG 973 kb) [file 12859_2017_1558_MOESM6_ESM.jpg]

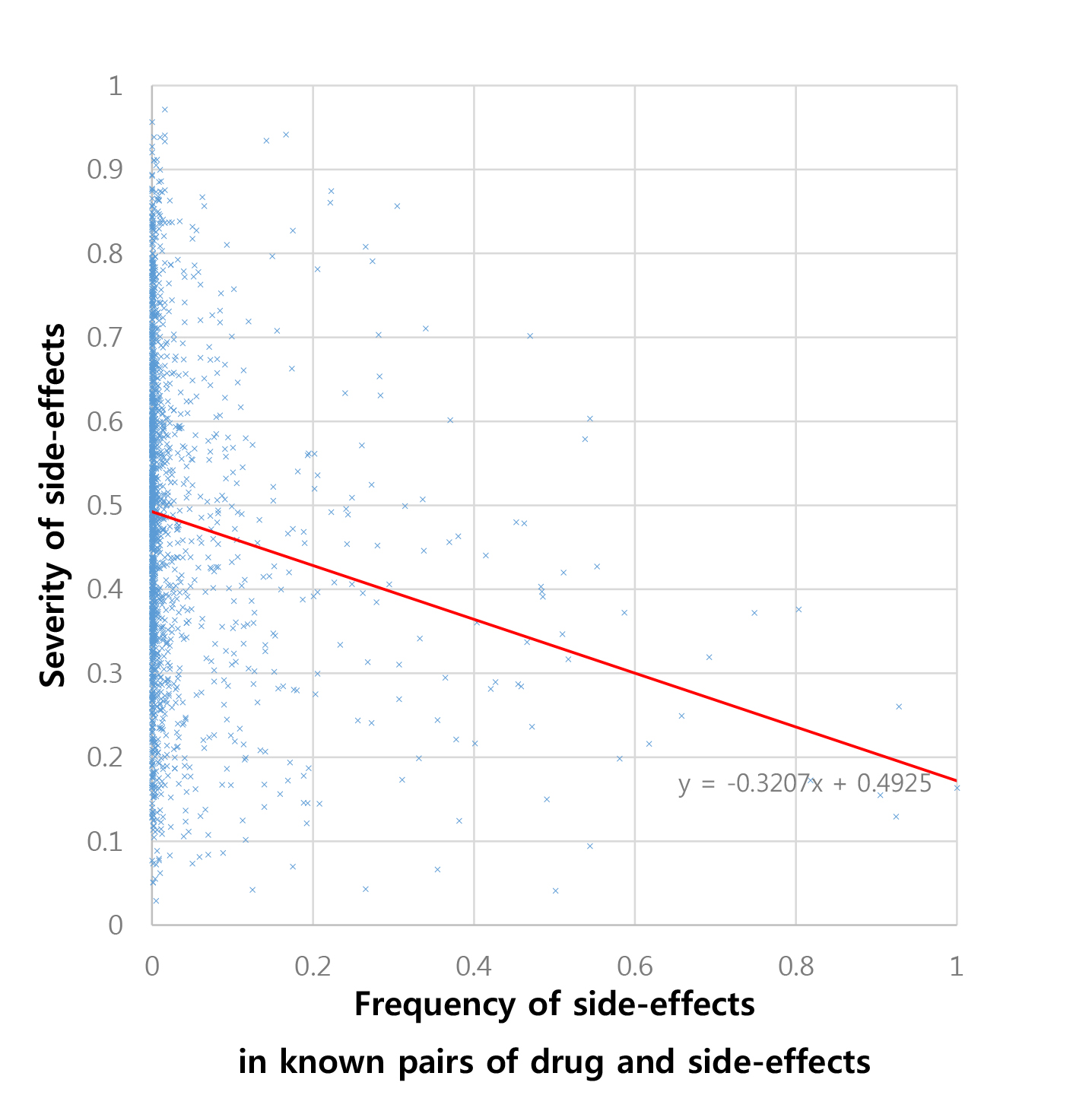

Supplement: Additional file 7: Figure S4. — The relationship between severity and frequency of the adverse reactions. Each point of the scatter plot represents a side effect. X-axis shows its frequency in known drug-side effect pairs and Y-axis displays the relative severity. (JPG 481 kb) [file 12859_2017_1558_MOESM7_ESM.jpg]
